# Endothelial Function and Carotid Intimal Medial Thickness in Asymptomatic Subjects With and Without Cardiovascular Risk Factors

**DOI:** 10.4021/cr194w

**Published:** 2012-07-20

**Authors:** Rajiv Ananthakrishna, Ravindranath K Shankarappa, Kapil Rangan, Dhanalakshmi Chandrasekaran, Manjunath C Nanjappa

**Affiliations:** a Department of Cardiology, Sri Jayadeva Institute of Cardiovascular Sciences and Research, Bangalore, India; b Department of Echocardiography, Sri Jayadeva Institute of Cardiovascular Sciences and Research, Bangalore, India

**Keywords:** Endothelial dysfunction, Carotid intimal medial thickness, Risk factors

## Abstract

**Background:**

The study was performed to assess endothelial function and carotid intimal-medial thickness (IMT) in asymptomatic patients, with and without risk factors for cardiovascular disease.

**Methods:**

A cross sectional survey of asymptomatic patients, aged 21 - 60 years, with and without risk factors for cardiovascular disease was recruited from the outpatient department of Cardiology. Endothelial function was evaluated by flow mediated dilatation (FMD) of the brachial artery and carotid IMT was determined using a high resolution B mode ultrasonography system.

**Results:**

A total of 104 patients were included in the study. The mean carotid IMT was 0.67 ± 0.05 mm in the group without risk factors and 0.78 ± 0.12 mm in the group with risk factors (P value < 0.05). Endothelial dysfunction (ED) and increased carotid IMT were more significant in the group with risk factors (P value < 0.001). Age, total cholesterol, LDL cholesterol, HDL cholesterol, triglycerides, blood pressure, body mass index and HbA1c had a significant correlation with both IMT and FMD response. A higher proportion of subjects with diabetes mellitus (87%), metabolic syndrome (86%) and family history of premature coronary artery disease (78%) had ED. In subjects with normal coronary angiogram, 71% had abnormal FMD response and 36% had increased carotid IMT.

**Conclusion:**

In asymptomatic subjects, risk factors for cardiovascular disease are significantly associated with objective evidence of ED and increased carotid IMT. FMD response and carotid IMT values are likely to yield additional information beyond traditional risk factors for classifying patients in regard to the likelihood of cardiovascular event. Therapeutic measures with the aim of improving endothelial function and reducing carotid IMT may reduce the burden of cardiovascular disease.

## Introduction

Atherosclerosis is an inflammatory disease that often begins in childhood and slowly progresses through a long asymptomatic phase. Endothelial dysfunction (ED) is an early marker of atherosclerosis and develops from the first decade of life, in the presence of genetic and environmental risk factors [1]. There is a high prevalence of premature coronary artery disease (CAD) in Asian Indians [2-4]. Hence, risk of cardiovascular events and effective preventive strategy are to be identified with greater sensitivity and urgency than clinical end points. The methodology for detection of pre-clinical atherosclerosis must be easy to perform, widely available and non-invasive. Endothelial function is an integrative maker of the net effects of damage from cardiovascular risk factors on the arterial wall, and can be assessed by non-invasive means. We postulate that asymptomatic patients with predisposition for cardiovascular events are likely to benefit from endothelial function assessment, which may assist in risk stratification. Hence, a cross sectional survey was undertaken to evaluate endothelial function in asymptomatic individuals. In addition to physiological assessment, carotid intimal-medial thickness (IMT) measurements as an additional surrogate marker of pre-clinical atherosclerosis were studied in the same cohort of patients.

## Methods

### Patient population

Subjects were recruited from the outpatient department of Cardiology, Sri Jayadeva Institute of Cardiovascular Sciences and Research, Bangalore, a tertiary care teaching hospital in South India.

#### Inclusion criteria

Individuals aged 21 - 60 years, with and without risk factors for cardiovascular events were enrolled in the study.

#### Exclusion criteria

1) Age ≤ 20 years and > 60 years; 2) Patients with acute coronary syndrome, stable CAD, peripheral vascular disease and cerebrovascular accident; 3) Prior history of revascularization (PCI/CABG); 4) Detection of plaques or stenosis in longitudinal B-mode imaging of carotid arteries; 5) Individuals on statins / angiotensin converting enzyme inhibitors / angiotensin receptor blockers / thiazolidinediones / nebivolol / estrogen containing preparations; 6) Presence of renal failure (serum creatinine > 1.4 mg /dL or estimated creatinine clearance < 60 mL/min).

CAD was excluded by a negative exercise stress test. Coronary angiogram (CAG) was performed in select cases with inconclusive stress test, to exclude CAD. Patients who qualify for the study were taken up for evaluation and details entered in a proforma.They were categorized into 2 groups-with and without risk factors for cardiovascular disease. Data analyzed include baseline characteristics (age, sex, body mass index, waist circumference, blood pressure, fasting blood sugar, post prandial blood sugar, HbAIc and lipid profile), flow mediated dilatation (FMD) of brachial artery and carotid IMT in all cases.

### Risk factors analyzed

Dyslipidemia, smoking, diabetes mellitus, hypertension, abdominal obesity, metabolic syndrome, family history of premature CAD (men < 55 years old, women < 65 years old) and physical inactivity were the risk factors evaluated in the present study. Dyslipidemia was diagnosed when any of the following abnormality was present: LDL-C > 140 mg/dL, HDL-C < 40 mg/dL and triglyceride > 150 mg/dL [5]. The diagnosis of metabolic syndrome was based on IDF 2005 criteria. Waist circumference ≥ 90 cm for men and ≥ 80 cm for women was the cut-off considered for obesity [6].

### Evaluation of FMD of brachial artery

The endothelial function was evaluated by ultrasonographic imaging of the brachial artery to assess endothelium dependent FMD [7]. After obtaining a rest image and measuring the baseline diameter, a blood pressure cuff was placed above the antecubital fossa and inflated to atleast 50 mmHg above systolic pressure. The cuff was deflated after 5 minutes. The post-stimulus diameter was measured 1 minute after cuff release. The brachial artery diameter was measured at the same time in the cardiac cycle, using ECG gating during image acquisition. FMD is expressed as the change in post-stimulus diameter as a percentage of baseline diameter. Normal response was defined as atleast 10% vasodilatation [8] and < 10% vasodilatation or paradoxical vasoconstriction was considered abnormal.

### Assessment of carotid IMT

The right and left carotid arteries were imaged using a high resolution B mode ultrasonography system. IMT (distance between intima-lumen interface and media-adventitia interface) was measured on the right and left common carotid arteries using IntiMaTe 2.0 Software Solution (Pixen Technologies Pvt. Ltd, Chennai, INDIA), at the distal 1 cm of the far wall [9]. The mean of ten IMT measurements (five from the left and five from the right) was used as the representative value for each subject. IMT values > 0.8 mm were considered abnormal [10, 11].

### Statistical methods

Discrete data are presented in the form of no. and percentage. Continuous data are presented in the form mean, standard deviation, minimum and maximum. Student’s t test or analysis of variance as appropriate was used for comparing mean values of selected variables in subjects with and without risk factors. Chi-square test (χ^2^) has been used to find out significant associations between discrete variables. A P-value of < 0.05 is considered to be significant.

## Results

Table 1 shows the clinical and biochemical features of the study groups. The mean age of subjects was 39.98 ± 11.18 years in those without risk factors and 42.55 ± 9.90 years in individuals with risk factors.

**Table 1:** Baseline Characteristics of Study Group (n = 104)

Variable	Without Risk factors (n = 50 )	With risk factors (n = 54)	P value
Age (years)	39.98 ± 11.18	42.55 ± 9.90	0.216
Male: Female ratio	1.38:1	1.84:1	-
BMI (kg/m^2^)	21.98 ± 1.29	24.45 ± 3.58	< 0.001
SBP (mm Hg)	109.32 ± 9.85	139.18 ± 15.55	< 0.001
DBP (mm Hg)	72.08 ± 5.75	87.03 ± 9.44	< 0.001
Creatinine (mg/dL)	0.89 ± 0.11	1.03 ± 1.10	0.347
TC (mg/dL)	168.06 ± 10.9	188.25 ± 25.95	< 0.001
LDL-C (mg/dl)	97.58 ± 11.0	117.47 ± 24.73	< 0.001
HDL-C (mg/dl)	43.82 ± 2.86	41.05 ± 3.84	< 0.001
TG (mg/dL)	133.24 ± 8.26	159.31 ± 78.45	< 0.001
Hb AIc (%)	5.69 ± 0.30	6.80 ± 1.04	< 0.001
FBS (mg/dL)	95.82 ± 7.20	112.25 ± 23.37	< 0.001
PPBS (mg/dL)	123.84 ± 8.21	177.35 ± 67.70	< 0.001

BMI, body mass index; SBP, systolic blood pressure; DBP, diastolic blood pressure; TC, total cholesterol; LDL-C, LDL cholesterol; HDL-C, HDL cholesterol; TG, triglycerides; HbAIc, glycosylated hemoglobin; FBS, fasting blood sugar; PPBS, post-prandial blood sugar. (All variables expressed as Mean ± SD).

Patients with risk factors had a higher body mass index, systolic blood pressure, diastolic blood pressure, fasting blood sugar, post-prandial blood sugar, HbA1c, total cholesterol, LDL cholesterol, triglycerides and lower HDL cholesterol. The risk factor profile is depicted in Table 2.

**Table 2:** Risk Factors (n = 54)

Risk factor	Number ( percentage )
Diabetes mellitus	23 (43%)
Hypertension	29 (54%)
Dyslipidemia	23 (43%)
Smoking	4 (7%)
Obesity	23 (43%)
Metabolic syndrome	14 (26%)
Physical inactivity	6 (11%)
Family h/o premature CAD	9 (17%)

ED and increased carotid IMT was more significant in the group with risk factors (Table 3, 4, 5) (P value < 0.001). The mean carotid IMT was 0.67 ± 0.05 mm in the group without risk factors and 0.78 ± 0.12 mm in the group with risk factors (Table 3) (P value < 0.05).

**Table 3:** FMD Response and Carotid IMT in Both Groups

Variable	Without Risk factors	With risk factors	P value
FMD Response (percentage change)	13.18 ± 2.38	6.53 ± 4.19	< 0.001
Carotid IMT (mm)	0.67 ± 0.05	0.78 ± 0.12	< 0.001

**Table 4:** FMD Response of the Study Population

Group	FMD of brachial artery		Total	χ^2^	P-Value
	Normal Response	Abnormal Response			
Without Risk Factors	46	4	50	41.948	< 0.001^*^
With Risk Factors	16	38	54		
Total	62	42	104		

*denotes significant difference. It is observed that there is a significant association between the FMD response and the risk factors (P < 0.001). Higher numbers of samples without risk factors have a normal FMD response whereas more number of samples with risk factors have an abnormal FMD response.

**Table 5:** Carotid IMT of the Study Population

	Carotid IMT		Total	χ^2^	P-Value
	Normal	Increased			
Without Risk Factors	48	2	50	7.400	0.007*
With Risk Factors	42	12	54		
Total	90	14	104		

*denotes significant difference. It is observed that there is a significant association between the IMT thickness and the risk factors (P < 0.001). Even though higher number of samples with and without risk factors have a normal IMT, more number of samples with risk factors have an increased IMT.

Table 6 presents the correlation of FMD response and IMT with the variables studied. Age, total cholesterol, LDL cholesterol, HDL cholesterol, triglycerides, blood pressure, body mass index and HbA1c had a significant correlation with both IMT and FMD response.

**Table 6:** Correlation of IMT and FMD With Variables

Factor	Carotid IMT		FMD	
	r	%	r	%
Age	0.651	< 0.001*	-0.320	0.001*
TC	0.525	< 0.001*	-0.557	< 0.001*
LDL-C	0.503	< 0.001*	-0.566	< 0.001*
HDL-C	-0.267	0.006*	0.341	< 0.001*
TG	0.289	0.003*	-0.278	0.004*
SBP	0.458	< 0.001*	-0.578	< 0.001*
DBP	0.552	< 0.001*	-0.604	< 0.001*
BMI	0.415	< 0.001*	-0.483	< 0.001*
HbA1C	0.614	< 0.001*	-0.651	< 0.001*

TC, total cholesterol; LDL-C, LDL cholesterol; HDL-C, HDL cholesterol; TG, triglycerides; SBP, systolic blood pressure; DBP, diastolic blood pressure; BMI, body mass index; HbAIc, glycosylated hemoglobin. *denotes significant correlation.

A higher proportion of subjects with DM (87%), metabolic syndrome (86%) and family history of premature CAD (78%) had ED (Table 7). In subjects with normal CAG, 71% had abnormal FMD response and 36% had increased IMT.

**Table 7:** Abnormal FMD Response and Increased Carotid IMT in Various Subsets

	Abnormal FMD response		Increased Carotid IMT	
	n	%	n	%
Diabetes mellitus	20	87%	9	39%
Hypertension	20	69%	9	31%
Dyslipidemia	17	74%	10	44%
Smoking	3	75%	1	25%
Obesity	15	65%	9	39%
Metabolic syndrome	12	86%	7	50%
Physical inactivity	2	33%	1	17%
Family h/o premature CAD	7	78%	2	22%
Normal CAG	10	71%	5	36%

## Discussion

Endothelium is a key regulator of vascular homeostasis. ED is well documented in adults with established atherosclerosis [12, 13] and is also equally important early event in subclinical atherogenesis [14, 15].The burden of established CAD on the individual and community is significant. Hence, it is important to have a window of opportunity to detect high risk patients in the preclinical phase of atherosclerosis and alter the natural history. In the present study, the impact of risk factors in asymptomatic subjects on arterial damage, were evaluated by FMD of brachial artery and simultaneous carotid IMT assessment. There are very few studies evaluating both parameters in the same cohort of patients. As mentioned previously, Asian Indians have a very high prevalence of premature coronary artery disease and in this context, studies of endothelial function and carotid IMT assume significance.

ED as assessed by FMD of brachial artery was significant in the subset of patients with risk factors. This highlights the need for management of not only the overt phase of atherosclerosis, but also pre-clinical disease. Similar observations suggesting ED as an early event in atherosclerosis in patients with risk factors were made in the studies by Celermajer et al [16] and Juonala et al [17]. There are two important observations in the present study-one relating to FMD response in individuals with family history of premature CAD and abnormal FMD response in patients with normal epicardial coronaries. Seven out of nine subjects (78%) with family history of premature CAD had abnormal FMD response. This is significant, as this is the only feasible method to assess subclinical atherosclerosis and cardiovascular risk in this subset of patients. A normal CAG does not exclude atherosclerotic burden and cardiovascular risk. In this study, more than two-thirds of individuals with normal epicardial coronaries and risk factors for cardiovascular disease had evidence of ED. There is a high probability that this subset of patients is denied the potential benefit of angiotensin converting enzyme inhibitors / angiotensin receptor blockers and statins, with the resultant risk of cardiovascular events in future. In the group without risk factors, 4 (8%) had an abnormal FMD response. This is most likely explained by the presence of unconventional and emerging risk factors and account for sudden cardiac arrest and acute coronary syndromes in apparently healthy subjects.

In addition, carotid IMT was significantly higher in the group with risk factors. Although, the number of subjects with increased IMT was less when compared to the number of individuals with abnormal FMD response in the same group with risk factors, it was statistically significant. It is likely that, with vascular damage, the physiological derangements precede anatomical changes. The absolute carotid IMT value is less in our study, as the population included younger adults and excluded subjects > 60 years. There is strong evidence that carotid IMT measurements can be used to indicate the degree of atherosclerotic burden and future cardiovascular risk, based on Rotterdam [18] and ARIC [19] studies. A small increase in mean carotid IMT of 0.2 mm is associated with an increase in relative risk for myocardial infarction and stroke of 33% and 28% respectively [10].

Our study highlights that age, lipid profile, blood pressure, body mass index and HbA1c have a significant correlation with both carotid IMT and FMD response. These are the same traditional risk factors for overt cardiovascular disease. This reinforces the fact that both endothelial function assessment and carotid IMT are useful markers of pre-clinical atherosclerosis and should be employed more often for optimal management of patients with risk factors.

### Study limitations

Exercise stress test has limitations of low sensitivity, and CAG was not performed in all cases. Psychosocial factors, dietary habits like consumption of fruits and vegetables, alcohol intake and non-conventional risk factors like lipoprotein (a), serum homocysteine, all of which influence the progression of atherosclerosis are not evaluated in the present study. There is no long term follow up, which is needed to establish the link between abnormal FMD response and CIMT to vascular events.

### Conclusion

In asymptomatic subjects, risk factors for cardiovascular disease are significantly associated with objective evidence of ED and increased carotid IMT, when compared to individuals without risk factors. In future, carotid IMT values and FMD response are likely to yield additional information beyond traditional risk factors for classifying patients in regard to the likelihood of cardiovascular event. Therapeutic measures with the aim of improving endothelial function and reducing carotid IMT may reduce the burden of cardiovascular disease.
